# Laparoscopic lavage: an option for surgical management of complicated diverticulitis

**DOI:** 10.1007/s00464-025-11617-4

**Published:** 2025-03-26

**Authors:** Héloïse Giron, Fabian Grass, Dieter Hahnloser

**Affiliations:** Department of Visceral Surgery, Lausanne University Hospital CHUV, University of Lausanne (UNIL), Rue du Bugnon 46, 1011 Lausanne, Switzerland

**Keywords:** Diverticulitis, Laparoscopic lavage, Technical description, Laparoscopy

## Abstract

**Background:**

Acute diverticulitis with perforation and peritonitis is a serious complication affecting up to 12% of patients. Peritonitis is classified into purulent (Hinchey III) or fecal (Hinchey IV) categories. The standard treatment has traditionally involved emergency surgery, such as bowel resection with or without anastomosis or Hartmann’s procedure, both of which carry high morbidity and mortality risks.

**Methods:**

In 2008, laparoscopic peritoneal lavage (LPL) emerged as a less invasive alternative for treating purulent peritonitis. This article outlines the LPL technique, emphasizing patient selection, procedural steps, and postoperative care.

**Results:**

Several clinical trials have compared LPL to traditional resection methods. These trials show that while LPL is associated with lower stoma prevalence and shorter recovery times, it also carries a higher risk of reoperation and misdiagnosis, especially in cases of fecal peritonitis. Proper patient selection, such as excluding immunosuppressed patients and those with Hinchey IV peritonitis, and careful intraoperative assessment are crucial for successful outcomes. While LPL is not superior to resection, it is a viable alternative in select cases.

**Conclusion:**

LPL offers a minimally invasive option for treating complicated diverticulitis in appropriately selected patients, though careful surgical expertise and patient-centered decision-making are essential to optimizing results.

**Supplementary Information:**

The online version contains supplementary material available at 10.1007/s00464-025-11617-4.

Perforation with peritonitis is a serious complication of acute diverticulitis, occurring in up to 12% of patients with diverticulitis [1]. The degree of contamination can be classified according to Hinchey into purulent (Hinchey III) or fecal (Hinchey IV) [2]. Historically, the standard approach has involved emergency surgery, typically consisting of bowel resection with or without primary anastomosis and diverting or end colostomy (Hartmann’s procedure) [1]. While resection procedures in the emergency setting carry a substantial risk of morbid mortality, a less invasive procedure has been described in 2008: laparoscopic peritoneal lavage (LPL) [3].

In this article, we describe the institutional technique of LPL for complicated diverticulitis and discuss indications, pitfalls, clinical, and long-term outcomes.

## Technical aspects

In our center, the discussion whether LPL should be performed is discussed with a senior staff board-certified colorectal surgeon and carried out by the on-call staff surgeon who is either properly trained and familiar with the procedure or under direct supervision by the colorectal surgeon.

The procedure begins with the placement under direct vision of the camera and two working trocars. The first step is an exploratory laparoscopy assessing all four quadrants of the peritoneal cavity. The primary goal consists of ruling out fecal peritonitis, an overt perforation or any other surgical pathology that may require more invasive surgery. In the presence of purulent (Hinchey III) peritonitis without any of the above findings, the pus is aspirated, and gentle blunt dissection with aspiration is performed to drain any loculated abscesses. The laparoscopic aspiration device is the preferred tool, which can be used as a blunt dissecting instrument, allowing for concomitant irrigation to mobilize gently bowel or omentum.

The entire abdominal cavity is then irrigated with sterile saline until the clear return of irrigation fluid, which in our experience typically requires at least 16 L. A critical aspect of the procedure is to avoid actively trying to localize the perforation. More specifically, dissection and mobilization of the colon, extensive lysis of firm adhesions and removal of the omentum from the suspected perforated area should be avoided. Of note, the omentum is gently mobilized and if mobile lifted but not actively dissected away from the suspected perforation site. Attempting to locate a hidden or sealed perforation could disrupt natural barriers and worsen the condition.

If an obvious perforation is identified during the procedure, it can be sutured to prevent further contamination or leakage providing the perforation is small (i.e., < 1 cm), there is no fecal contamination or spillage and bowel edges look healthy and well perfused. However, larger overt perforations warrant a change of strategy including bowel resection. After completion of the lavage, two large caliber drains are placed as sentinel devices to allow continuous drainage of any residual fluids to prevent further infection or abscess formation.

The technique described above is related to our institutional experience. There are no specific recommendations regarding the number of drains or the required volume of lavage. These decisions are adapted according to disease presentation and thus case- and surgeon-dependent. However, we typically suggest placement of at least two medium-size Jackson-Pratt (JP) drains.

To guide postoperative care, the patient’s clinical condition represents the most reliable surveillance tool. We suggest close monitoring of vital parameters including hemodynamics and body temperature along with inflammatory markers and close surveillance of the aspect of the drained fluid. An initial increase in inflammatory parameters is expected during the first 48 h after surgery, with a significant reduction after 3–4 days. Hence, we suggest performing a C-reactive protein (CRP) level at the 3rd postoperative day (POD). Persistent fever, unchanged or elevated inflammatory markers beyond POD 3, or increasing pain are key indicators to repeat imaging or surgery with a low threshold. If the imaging shows only an abscess, radiological drainage is performed. Surgical revision is carried out in case of persistent peritonitis with free fluid, to offer surgical resection considering LPL management failed. We do not perform a second lavage. Importantly, the decision must be individualized for each patient and clinical situation. Hence, a standardized algorithm cannot be established. The drains are removed on POD 5, irrespective of the drainage rate. A close outpatient follow-up visit is scheduled within 10 days of discharge. Large spectrum antibiotics are continued for 10 days in accordance with the institutional recommendations in case of peritonitis, which is slightly longer than the suggested 5–7 days described in a recent review [4].

## Discussion

Three major randomized controlled trials (RCT) were conducted comparing laparoscopic lavage with sigmoidectomy. The LADIES trial was a multicenter RCT from Belgium, the Netherlands, and Italy, published in 2015 was split in two groups [5]. The first one was the DIVA group comparing Hartmann’s procedure and sigmoidectomy plus primary anastomosis. The second was the LOLA (LaparOscopic LAvage) group comparing laparoscopic peritoneal lavage to sigmoidectomy. In the LOLA group, diagnostic laparoscopy was performed to confirm the diagnosis and randomize patients. Only patients with purulent (Hinchey III) peritonitis without overt perforation were assigned to either LPL or sigmoidectomy with or without primary anastomosis. Routine sigmoidectomy was not recommended for patients after LPL. Main exclusion criteria were patients with fecal peritonitis, those who were hemodynamically unstable, and those receiving steroids. The primary outcome measure was a composite endpoint assessing major morbidity and mortality within 12 months post-treatment. The LOLA group of the study was terminated early for safety reasons after the 3rd planned interim analysis [5], revealing that sepsis control was not achieved in 24% of cases in the LPL arm, primarily due to misdiagnosed fecal peritonitis resulting from insufficient surgical exploration in most cases. This study highlights thus the critical importance of thorough intraoperative assessment to ensure accurate diagnosis and appropriate management. Notably, salvage surgery remained a viable option in all instances where initial treatment failed, underscoring the adaptability of surgical intervention in these complex cases. In conclusion, the study did not demonstrate a superiority of lavage compared to primary resection.

Three-year follow-up of the LOLA group showed that morbidity and mortality were comparable in patients who had undergone LPL or sigmoidectomy [6]. However, there were less definitive stomas and a lower reoperation rate in the LPL group. This aspect is of particular importance. In our experience, patients have a negative perception and reluctance regarding ostomy creation. Being able to offer a surgical alternative after in-depth discussion of expected benefits and risks is thus important for our practice.

The author group of the LADIES trial further compared the cost-effectiveness of LPL compared to sigmoidectomy [7]. The comparison over a year revealed lower overall costs in the LPL group. Despite the higher probability of additional interventions in the LPL group including drainage procedures, the less invasive and resource-consuming LPL strategy resulting in a shorter hospital stay contributed to cost savings. Even after performing sensitivity analyses with varying cost estimates, the differences in favor of LPL remained consistent. This perspective appears interesting since offering a more affordable treatment alternative. However, whether this cost advantage persists when comparing LPL to primary anastomosis and loop ileostomy represents an ongoing matter of debate.

The SCANDIV trial (Scandinavian Diverticulitis trial) is another key study comparing LPL (101 patients) with primary resection (98 patients) for patients with perforated and purulent peritonitis (Hinchey III) [8]. The primary outcome of the SCANDIV trial was severe postoperative complications (Clavien-Dindo > IIIa) within 90 days, revealing no difference between the two groups (31% vs. 26%, respectively). However, the reoperation rate was higher in the LPL group (20% vs. 6%, respectively), while operating time was shorter, with no differences in length of stay or quality of life. The study further described 4 missed sigmoid carcinomas in the LPL group, further highlighting that cancer may be hidden in the inflamed colonic segments in up to 8% of patients [9]. Taken together, the SCANDIV trial did not support LPL as alternate treatment over sigmoidectomy for perforated diverticulitis. However, a more recent 5-year follow-up analysis showed no differences in severe complications, even though 30% of patients in the LPL group eventually underwent sigmoid resection, mainly for disease recurrence [10]. Furthermore, the stoma prevalence of 8% was significantly lower in the LPL group compared to the resection group (33%), suggesting shared decision-making considering not only short- (complications, reoperation) but also long-term (stoma prevalence) consequences.

Finally, the 2016 DILALA trial, which compared laparoscopic lavage and Hartmann’s, found encouraging outcomes after LPL in the 12-week postsurgical observation period [11]. While morbid mortality and reoperation rate did not differ between the two groups, LPL was associated with shorter operating time, shorter time in the recovery unit, and shorter length of hospital stay. This trial hence supported the idea of LPL as an effective, less invasive, and safe alternative to Hartmann’s, while emphasizing the need for careful patient selection and monitoring to prevent complications such as recurrent infection or sepsis. These considerations were also supported by a meta-analysis of the same group [12].

Regarding disease recurrence after successful LPL in Hinchey III peritonitis, a recent retrospective series revealed a recurrence rate of 42% after a median follow-up of 63 months [13]. This further animates the debate whether elective resection after LPL should be routinely performed.

Recently published European guidelines do not recommend LPL as a first-choice treatment, however acknowledging its role as a valid and safe treatment option in selected patients [14]. However, American guidelines do not recommend LPL in any case [15].

## Conclusion

The present dynamic manuscript aimed to showcase the proper technique of LPL as an alternative to resection procedures. Key principles include proper patient selection (exclusion of immunosuppressed patients and patients with Hinchey IV peritonitis) and the presence of surgeons properly trained and familiar with the technique to ensure adequate decision-making.

In the era of patient-reported outcome measures (PROMs), treatment goals need to be discussed and tailored to the individual patient. Considering these principles, LPL should be part of the repertory of surgical treatment strategies in the case of complicated diverticulitis.

## Supplementary Information

Below is the link to the electronic supplementary material.

Supplementary file1 (MP4 451628 KB)
